# Is Resynchronization Pacing Proarrhythmic Among Congenital Heart Patients? An Evaluation and Review

**DOI:** 10.3390/jcdd13030117

**Published:** 2026-03-04

**Authors:** Peter P. Karpawich, Kathleen Zelin, Corinne Biggs, Swati Sehgal, Jennifer Blake, Chenni Sriram, Pooja Gupta

**Affiliations:** Section of Cardiology, Departments of Pediatrics, The Children’s Hospital of Michigan, Central Michigan and Wayne State Universities Schools of Medicine, Detroit, MI 48201, USA; kzelin@dmc.org (K.Z.); cbiggs@dmc.org (C.B.); ssehgal@dmc.org (S.S.); jblake2@dmc.org (J.B.); csriram@dmc.org (C.S.);

**Keywords:** cardiac resynchronization pacing, heart failure, arrhythmias, congenital heart disease, Pediatric Cardiology, Adult Congenital Heart, heart transplant

## Abstract

Background: Cardiac resynchronization therapy (CRT) can be an effective form of heart failure (HF) management among congenital heart disease (CHD) patients (pts) with and without surgically repaired defects. However, very long-term results are limited. Recently, CRT has been implicated to be proarrhythmic among older CRT recipients. This issue is largely unknown among younger CHD-CRT pts. This study presents up to a 20-year (y) continuous review of any arrhythmia (Arr) burden associated with CRT among CHD-HF pts. Methods: From 1999 to 2024, outcomes of 45 CHD-HF pts (NYHA II-IV) (age 4–57 y [mean 26]; 31% female) were compared between those on established medical management (MM) (*n* = 18) and CRT recipients (*n* = 27) followed continuously for 1–20 years. Pre-existing and any de novo Arr that occurred following CRT were documented. Clinical assessments were continuous. Results: Follow-up was for 1 to 20 y (mean 7.5 y ± 0.7 sem). Patient demographics (CRT vs. MM groups) were comparable. Pre-existing Arr were found in 16 pts (38%) from both groups: accelerated junction (one CRT), atrial flutter (one CRT; two MM), and ventricular tachycardia (six CRT; six MM). During follow-up, outcomes included 14 pt deaths and 7 heart transplants (HTs). Of these, pre-existing Arr were causative among three CRT recipients: two ≤ 2 y and one > 5 y after CRT. There were no new-onset Arr in any pt groups. CRT significantly improved patient survival free from HT or death at 10 y (44 vs. 13% [*p* < 0001]). Conclusion: When applied effectively, CRT benefits CHD-HF pts without causing additional arrhythmias. However, pre-existing Arr remain a concern reflecting persistently adverse intrinsic myocellular issues among CHD-HF pts.

## 1. Introduction

With surgical advances over the past several decades, life expectancies of children born with congenital heart disease (CHD) have increased. However, a repaired congenital heart does not equate to a normal heart. Due to intrinsic cardiac anatomies and surgical repairs, clinical outcomes often include morbidities of early-onset heart failure (HF) and arrhythmias (Arr) [1,2]. Since listing for a heart transplant (HT) does not assure organ receipt, improving function and delaying HT becomes an important factor in clinical care [3].

Cardiac resynchronization pacing therapy (CRT) can be an effective HF intervention to improve contractility as well as aid in arrhythmia management, as attested by multiple clinical studies, including pts with surgically repaired congenital heart defects [4,5,6,7]. However, in deference to the 2023 HRS Guidelines on physiologic pacing, targeted and specific lead-location implant has typically not been followed [8,9,10]. As a result, pacing initiated from any two separate ventricular sites, as frequently performed, although constituting “biventricular pacing”, does not always result in effective contractility resynchronization improvements and cellular remodeling. This has resulted in the reported lack of clinical CRT benefits with “non-responder” rates ranging from 30 to 90% of CRT recipients as well as associated adverse myopathic effects. As a result, a newer and somewhat technically easier left bundle area pacing approach is now advocated for some patients [11,12,13,14,15,16]. In this regard, as CRT applications are scrutinized, concerns have arisen that this therapy, per se, may be proarrhythmic [17,18]. This is especially disconcerting as Arr are a well-recognized aspect of CHD, either due to intrinsic anatomy, altered hemodynamics or surgical repairs [19,20]. Any proarrhythmic contributions by CRT itself, therefore, may adversely influence decision-making.

As a result, the specific role of CRT in either inducing or worsening pre-existing Arr among this subset of CHD-HF pts remains largely unknown. To our knowledge, this report presents the first continuous and very chronic (20 years) clinical outcome analysis, as well as a literature review, of CRT-related new or worsening arrhythmias among CHD-HF CRT recipients.

## 2. Methods

This is an analysis of the chronic efficacy of CRT vs. standard pharmacologic heart failure (HF) medical management (MM), with an emphasis on changes in arrhythmia (Arr) burdens (de novo onset or worsening of existing Arr), for all CHD heart failure patients ≥ 4 years of age being evaluated for heart transplant listing. The study was performed at the Section of Cardiology as part of the Children’s Hospital of Michigan’s Heart Failure (HF) and Adult Congenital Heart Disease (ACHD) Programs. The study was approved by the Institutional Review Board. As is typical of many HF/ACHD Programs, patients included those with diverse structural congenital cardiac defects, both before and following any surgical or other interventions, as well as those with congenital heart block and cardiomyopathies. All exhibited clinical HF (NYHA II–IV) on appropriate MM (preload, afterload and inotropic supportive agents) from 1999 to 2025 as part of the standard institutional HF management protocols. Patients with known conduction issues and Arr had appropriate implantable pacing devices (pacemakers and/or ICDs) as well as anti-arrhythmic medications as clinically indicated. Referral for possible CRT consideration as additional HF management was based on the discretion of the individual patient’s physician, patient interest and an NYHA status ≥ II. Based on established institutional selection criteria for pre-HT listing, referred patients underwent noninvasive testing with a standard noninvasive ECG and echocardiogram with Doppler evaluations. In addition, all also underwent a standard hemodynamic cardiac catheterization procedure to directly evaluate pressures and contractility indices. To assure CRT effectiveness prior to implant, patient selection was dictated on pre-implant measured contractility (dP/dt-max) responsiveness to acute biventricular pacing (BVP) with mapping of various targeted lead site locations. The most optimal sites were marked fluoroscopically and leads inserted to approximately those locations as previously reported [21]. In this manner, all leads were inserted to assure optimal acute CRT-paced responsiveness. Patients exhibiting a ≥15% improvement in directly measured contractility indices during acute BVP were given the option of undergoing either a de novo implant or a CRT upgrade of an existing pacing system. Those with a <15% improvement indicated that effective CRT pacing was not attained and those pts were continued on appropriate MM pharmacotherapies.

**Figure 1:**
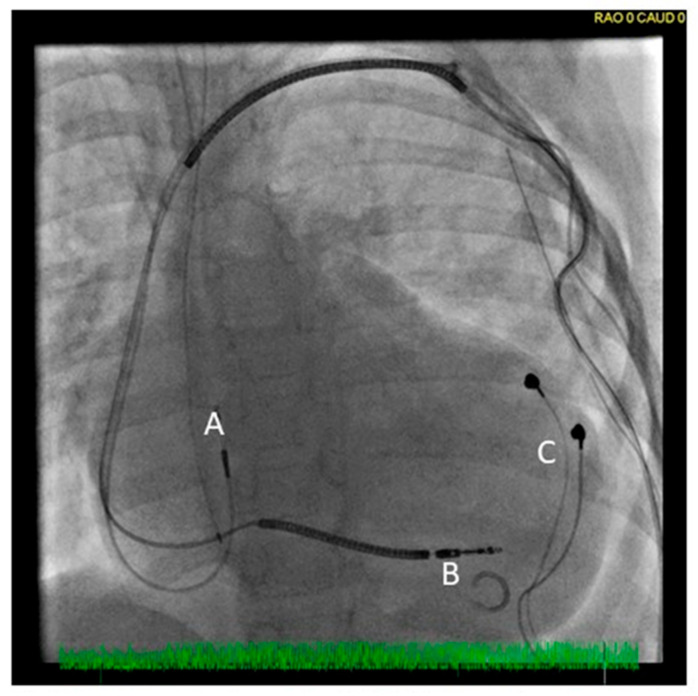
Fluoroscopic view of a CRT-ICD in a 4 y female with dilated cardiomyopathy, heart failure and ventricular arrhythmias. A hybrid transvenous (A: right atrium; B: right ventricle) and LV epicardial lead (C) system is illustrated. Leads are tunneled to an abdominal generator.

All device implants/upgrades were performed using standard surgical techniques under fluoroscopic guidance with lead threshold/sensing measurements by established implanters following the protocol. Depending on the CHD anatomy, CRT pacing was achieved either by a de novo device implant with both septal and coronary sinus leads or, if pts had pre-existing ventricular pacing, with the addition of a second ventricular lead. This entailed either epi- or endocardial leads depending on the CHD (e.g., an additional transvenous left ventricular [LV] lead among pts with Mustard/D-transposition of the great arteries with an existing epicardial right [R] V lead). In this manner, a “hybrid” lead approach of combined epi- and endocardial lead implants was applied as determined by patient age or anatomy (Figure 1). Patients with single (S) V anatomy underwent intraoperative implant of two opposed epicardial V leads to achieve dual-site ventricular pacing. Among pts with pre-existing ventricular Arr, a CRT-D system was implanted. Any anti-arrhythmic medications were continued as clinically indicated.

Following the initial noninvasive and hemodynamic contractility assessments, all patients were continually followed in an outpatient setting to evaluate their respective conditions (MM or CRT). This included clinical assessments with the recording of NYHA status, results of echocardiograms, ECGs, any device interrogations, and data from any additional cardiac catheterization studies. There was specific attention paid to the determination of the persistence of any pre-existing or new-onset arrhythmias at each evaluation. Individual patient follow-up was continuous with clinical outcomes recorded for the duration that each patient was followed. Once any patient underwent HT or incurred death for any reason, follow-up was concluded and the cause was recorded.

## 3. Statistics

All clinical data was reported as mean ± SEM for continuous variables and frequency for categorical variables. Patient group data was compared using the unpaired Student *t*-test and Chi Square analysis using Graph-Pad Prism software (GraphPad Prism Software, version 7; La Jolla, CA, USA). Statistical significance was defined as a *p* valve ≤ 0.05.

## 4. Results

From 1999 to 2024, 45 pts (ages 4–57 y [mean 26 y]); 38% female) over 4 years of age, with/without repaired structural CHD defects and NYHA II-IV status, were referred for possible CRT as adjunct HF therapy in the Congenital Heart Failure clinic at our institution. These pts constitute the study population. Table 1 illustrates patient demographics and age at the time of initial evaluation as well as individual patient follow-up durations. As arrhythmias can be related to altered echocardiographic and hemodynamic measurements, as well as any pre-existing pacemakers, these variables were compared. In addition, any known arrhythmias are indicated. Patient cardiac anatomies were variable as expected and included a systemic RV as found in D-TGA/Mustard repairs *(n* = 13) and SV (*n* = 4). The remainder had normal ventricular anatomical relationships associated with various CHD surgeries (including septal defects and Tetralogy of Fallot), congenital atrioventricular block (CAVB) or infantile/hypertrophic cardiomyopathies that had progressed to dilated cardiomyopathy (DCM). Specific pre-existing arrhythmias (atrial flutter [AF] *n* = 3, junctional tachycardia [JT] *(n* = 1) and monomorphic ventricular tachycardia [VT] *n* = 12) were identified in 16 pts (36%) as indicated. All of these pts were treated with anti-arrhythmic medications and none had any prior aborted sudden death episodes. Twenty-eight pts (62%) had pre-existing ventricular pacing (VP) with/without ICD capability. Of these, 26 were dual-chamber systems.

All pts who exhibited the established ≥15% acute contractility improvement with temporary BVP agreed to and underwent a CRT implant/upgrade. This amounted to 27 pts (60%) and constituted the CRT Group. The remaining 18 pts who did not meet the CRT-implant patient selection criteria continued on standard medical management protocols (MM Group) as a non-CRT control group. Baseline comparisons between the two patient groups are shown in Table 2. As indicated, except for age and the presence of repaired congenital defects, there were no differences in pre-existing arrhythmias or pacing, echocardiographic and hemodynamic measurements between groups.

**Figure 2:**
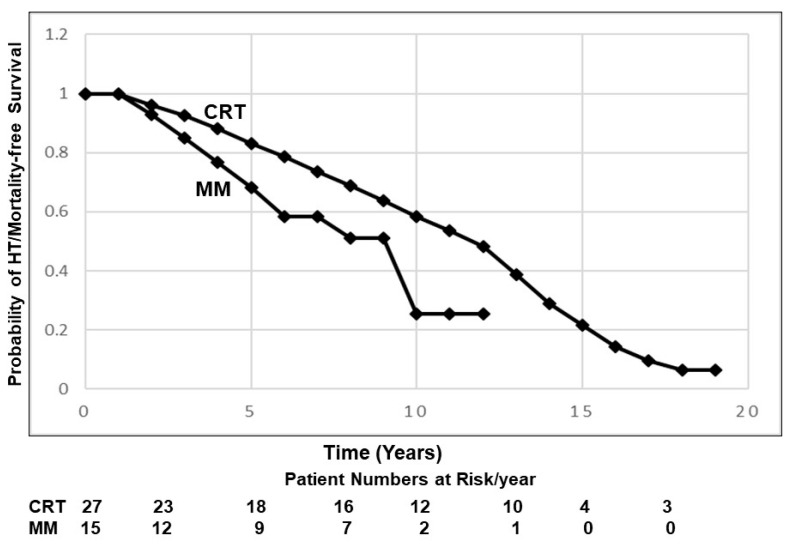
Kaplan–Meier graphs of survival freedom from death or heart transplant among all patients, separated between those with CRT compared with MM over the 20 y study interval. As illustrated, although early (<5 y) survival results were relatively comparable, long-term follow-up (>10 y) demonstrated a significant (*p* < 0.05) survival benefit with CRT.

Among all pts, the continuous patient follow-up duration ranged from 1 to 20 years (mean 7.5 y ± 0.7 sem) with any cause of death, HT or current clinical status recorded at last follow-up. There were comparable mean follow-up intervals between groups (CRT: 8.5 y ± 1.0; MM 6.0 y ± 0.8 [NS]). Kaplan–Meier survival curves are illustrated in Figure 2. There were no new-onset arrhythmias identified in any patient during their entire respective follow-up evaluations, even among those who died or whose clinical deterioration required transplant. Among CRT recipients, identified pre-existing Arr contributing to HT or mortality occurred in only three pts (11%). Two (pts #1 and 7) experienced worsening and sustained VT episodes requiring cardioversion ≤ 2 years following CRT-D implant, leading to early HT. A third patient (#25, age 38 y, D-TGA/Mustard), improved clinically after CRT-D implant and was de-listed from HT. However, 6 years later, heart failure worsened. Although re-listed for transplant, he experienced an incessant VT episode that was not terminated by appropriate ICD discharges. The remaining three pts with pre-existing VT remained asymptomatic at 3, 8 and 20 years post CRT. All exhibited arrhythmia stability. An additional nine pts died or underwent HT, due to progressive HF or non-cardiac causes. Among the MM group pts, progressive heart failure, in some instances due to poor medical compliance, not arrhythmias, was the identified reason for HT or death in eight pts, including two pts with a history of VT. All other pts with pre-existing arrhythmias remained clinically stable on medical therapy. No ventricular assist devices were implanted.

## 5. Discussion

In contradistinction to CRT among more elderly pts with normal cardiac anatomy, the recently published 2023 Heart Rhythm Society Guidelines provides only a maximum class of recommendation (COR) of “2a” and LOE “C-LD” for the application of CRT for CHD and pediatric HF pts, attesting to the limited available data on its actual clinical efficacy [8]. This is primarily due to the relatively small number of reported instances of CRT applications in this very diverse patient population associated with imprecise as well as inconsistent definitions of success, often with relatively short follow-up intervals.

Arrhythmias are well-recognized among HF pts, being associated with a poor prognosis [22,23]. Comparably, they are also common among CHD even without HF. Therefore, the recent implication that CRT, per se, can be proarrhythmic potentially reduces or negates benefits, especially to younger CHD-HF patients. Unfortunately, arrhythmias following CRT, and their respective clinical implications, have only infrequently been included among many previous pediatric/adult CHD-HF CRT studies [24,25,26,27,28,29,30,31]. Although these studies have contributed pertinent clinical information and have advanced knowledge of CRT applications among this diverse patient subset, the lack of specifically addressing existing or de novo arrhythmias limits understanding of potential long-term CRT efficacy. The absence of this information has somewhat been attributed to study design protocols and data collection [25]. By specifically addressing existing and any de novo arrhythmias, this current study helps to fill the void in current knowledge of risk/benefits of CRT among CHD-HF patients.

With improved CRT-modified hemodynamics, pre-existing Arr might be expected to lessen. However, results have not been consistent [32,33,34]. Potential explanations include ineffective remodeling to direct pacing-induced adverse transmural activation sequences [7,14,35]. There are multiple studies indicating patient criteria (e.g., low EF and QRS morphology) to support CRT application in general. However, recommendations of how to optimize resynchronization with specific lead locations have not been well established, even though targeted lead implants have been advised [9,10]). As a result, leads are frequently placed empirically in the coronary sinus and right ventricle without actually evaluating mechanical ventricular contractility, contributing to the highly reported “non-responder rates”. The positive response of patients in this study attest to the benefits of preselecting lead implants and optimizing contractility. Pacing-induced myocellular remodeling is not instantaneous and currently requires an unknown time duration following the more acute change in contractility vectors, such as those measured by ejection fraction [13,34,35,36,37,38]. This concept is applicable when abnormal rhythms occur soon after CRT implant, such as in the two CRT recipients in this study that experienced apparent early clinical improvement in measured contractility variables but worsening Arr requiring HT ≤ 2 years later.

In this study, there were minimal contributions from pre-existing Arr leading to HT or mortality regardless of patient group, attesting to improved HF therapies in the current era. Of major importance is that CRT pacing, per se, did not increase any Arr burden in any patient. However, intrinsically altered myocellular architecture due to CHD anatomy and/or surgeries may persist in spite of initial apparent clinical improvement following CRT. This may continue to be responsible for late-onset adverse rhythms. As indicated in this study, one patient with CRT-D and a systemic right ventricle on anti-arrhythmic therapy experienced refractory VT non-amenable to defibrillation 6 years following initial clinical HF improvement. This somewhat coincides with a previous CHD-CRT study that indicated VT occurring in an apparent effectively CRT-treated patient several months after implant [27].

Although non-specific markers of contractility, such as EF or QRS morphology, may show improvement, the real test of CRT efficacy still requires evaluation at the cellular level. Since CRT success remains poorly defined, a better analysis of biomarkers with a focus on cell metabolism has now been advocated, attesting to the need to recognize intrinsic myocellular changes associated with altering paced contractility vectors [4,39]. Such a recognition may better optimize patient selection as well as provide a more accurate definition of CRT responsiveness [40,41]. Future studies will be required to fully determine these concepts.

## 6. Limitations

As a single institutional study, patient numbers are not as large as found in multisite review studies. That does fit with the fact that CRT applications for heart failure among children and CHD patients has been limited when compared with the elderly. Guidelines for Pediatric/CHD implant and patient selection are also less well-defined. In that manner, any CRT application among CHD patients will inherently have a potential selection bias. However, the study does represent a typical distribution of patients seen among Pediatric and Adult Congenital Heart HF centers and follows the recommended targeted lead implant that achieves optimal contractility response. In addition, it presents the first and longest continuous clinical follow-up study to date among CHD pts with an emphasis on contributions, if any, to existing or denovo arrhythmias with eventual outcomes. The inclusion of comparable patient ages, pre-existing pacemakers and arrhythmias, CHD anatomies, and hemodynamic variables allows for a ready determination of actual therapeutic clinical efficacy of CRT.

## 7. Conclusions

CHD pts are a very diverse population with the potential for early-onset HF and a predisposition to arrhythmias, often based on anatomy and surgical repairs, and patient selection guidelines are limited. However, with proper application of the preselection of patients based on contractility responses to targeted lead implant, CRT can add very long-term clinical benefits among CHD-HF patients. Importantly, such effective CRT, per se, does not appear to contribute to any de novo arrhythmias. However, an understanding of chronic myocellular/metabolic changes following CRT is still required. Positive myocellular remodeling may be a time-variable response and may not always improve following apparent acute contractility changes detected by noninvasive measurements. Persistence of adverse underlying myocardial substrates causing arrhythmias due to CHD and surgical interventions remain a concern for long-term clinical CRT efficacy.

## Figures and Tables

**Table 1:** Comparative patient demographics and outcomes at last follow-up.

CHD	Age	Gender	LVEF	LVEDD	VEDP	dP/dt	QRS	Pre V Pace	F/Up	Outcome	Arrhy	CRT-D
	Years		%	cm	mmHg	mmHg-s	ms		Years			
DCM	4	F	29	5.9	16	483	160 *	yes-D	1	HT	VT	yes
CAVB	13	F	25	5.8	14	625	161 *	yes-D	12	NYHA 1-2		
CAVB	14	F	43	5.4	9	647	134 *	yes-D	20	NYHA 1-2	VT	yes
DCM/WPW	14	M	26	7.7	21	581	172	no	4	HT		
CAVB	14	M	25	6.9	15	332	147 *	yes-D	18	NYHA 1-2		
DCM	15	M	65	4.8	10	735	136 #	no	5	died ^		
HCM-DCM	18	M	45	5.4	17	603	190 *	yes-D	2	HT	VT	yes
AS/coarct	18	M	29	5.5	17	774	94	no	14	NYHA 1-2	VT	yes
AV Canal	19	F	25	6.7	19	188	166 *	yes-D	6	HT		
CAVB	20	F	10	8.1	22	264	210 *	yes-V	3	died-HF		
DCM	20	F	15	6.2	17	637	120	no	18	NYHA 1-2	JT	
CAVB	21	M	40	8.1	28	454	160 *	yes-D	4	died-HF		
CAVB	21	F	28	5.8	14	827	168 *	yes-D	9	NYHA 2		
TOF	23	M	35	6.4	15	542	172	no	5	died ^		
L-TGA/VSD	24	M	NA	5.9	23	490	160 *	yes-D	12	HT		
D-TGA/Mustard	24	M	NA	NA	8	735	150 *	yes-V	12	NYHA 1-2		
DILV/Fontan	27	F	35	4.4	14	768	176 *	yes-D	16	NYHA 2		
L-TGA	27	M	NA	6.9	15	674	164 *	yes-D	12	NYHA1-2		
D-TGA/Mustard	27	M	NA	NA	6	647	95	yes-D	10	NYHA 2-3		
AV Canal	28	F	14	5.1	10	618	120 *	yes-D	2	died ^		
D-TGA/Mustard	29	M	NA	NA	12	769	157	no	12	NYHA 1-2		
DILV/Fontan	29	F	NA	NA	10	560	122	no	7	NYHA 2		
TOF	31	M	40	7.1	12	671	172	no	3	NYHA 1-2	VT	yes
DORV/VSD	32	M	NA	5.2	22	800	202	no	2	died-HF	AF	
D-TGA/Mustard	32	M	NA	NA	18	413	208 *	yes-D	6	died-VT	VT	yes
D-TGA/Mustard	38	M	NA	NA	8	530	150	no	11	NYHA 2		
D-TGA/Mustard	39	M	NA	NA	11	840	157 *	yes-D	4	died ^		
**mean ± sem**	**23 ± 1.6**	**37%F**	**31 ± 3.2**	**6.2 ± 0.2**	**14.9 ± 1.0**	**600 ± 32.3**	**156 ± 5.7**	**17 (63%)**	**8.5 ± 1.0**		**30%**	
**CHD**	**Age**	**Gender**	**EF**	**LVEDD**	**VEDP**	**dP/dt**	**QRS**	**Pre V Pace**	**F/Up**	**Outcome**	**Arrhy**	**ICD**
	**Years**		**%**	**cm**	**mmHg**	**mmHg-s**	**ms**		**Years**			
AS	9	F	40	6.4	7	500	180 *	yes-D	2	HT		
TOF	13	F	30	6.1	24	823	200 *	no	7	HT		
DCM	15	F	24	6.3	14	600	100	no	9	NYHA2-3	VT	yes
HLHS	16	M	NA	NA	10	590	120	yes-D	1	died-HF	VT	yes
TOF	17	M	35	6.6	8	698	157	yes-D	9	NYHA 2		
DCM	18	F	17	5.3	14	600	96	no	1	NYHA 2-3	VT	yes
ASD	28	M	36	7.7	14	696	150 *	yes-D	9	died-HF		
D-TGA/Mustard	28	M	NA	NA	15	798	128 *	yes-D	3	died-HF		
D-TGA/Mustard	29	M	NA	NA	31	828	90	no	13	NYHA 1-2		
DCM	30	M	32	5.5	11	766	120	no	11	NYHA 1-2	VT	yes
D-TGA/Mustard	32	M	NA	NA	17	640	127 *	yes-D	5	died-HF		
D-TGA/Mustard	35	M	NA	NA	19	605	126	no	7	NYHA 1-2	VT	yes
TOF	36	M	48	4.1	12	522	196 *	yes-D	2	NYHA 2	AF	yes
Truncus/VSD	37	M	40	6.3	6	432	200 *	yes-D	4	died-HF		
DILV	40	F	NA	NA	8	698	60	no	5	NYHA 2-3		
Pulm Atr/VSD	41	F	46	3.6	13	833	176 *	yes-D	3	NYHA 2-3	AF	
D-TGA/Mustard	45	F	NA	NA	11	850	180 *	yes-D	10	NYHA 1-2		
TOF	57	F	30	4.1	15	732	120 *	yes-D	7	died-HF	VT	yes
**mean ± sem**	**30 ± 3.3**	**44%F**	**32 ± 2.8**	**5.6 ± 1.2**	**14 ± 1.4**	**678 ± 29.5**	**140 ± 9.8**	**11 (61%)**	**6.0 ± 0.8**		**44%**	

Legend: A: CRT; B: MM. Age: patient age at study onset; Arrhy: pre-study existing arrhythmias; AF: atrial flutter; AS: aortic stenosis; AV: atrioventricular; CAVB: congenital atrioventricular block; CHD: congenital heart disease; D: dual-chamber paced; DCM: congenital dilated cardiomyopathy; DILV: double inlet LV; DORV: double-outlet right ventricle; dP/dt: pressure/time contractility: D-TGA: dextro-transposition of the great arteries; HF: heart failure; HT: heart transplant; HLHS; hypoplastic left heart syndrome; JT: junctional tachycardia; L: levo; LVEDD: left ventricular end-diastolic diameter; NA: data not available; NYHA: New York Heart Association; TOF: Tetralogy of Fallot; VEDP: ventricular end-diastolic pressure; VSD: ventricular septal defect; VT: ventricular tachycardia. Symbols: * paced QRS, # intrinsic LBBB; ^ non-cardiac cause of death. Bold type: accentuates arrhythmias and causes of death.

**Table 2:** Comparative demographics including pre-existing arrhythmias, pacing, ECG, and echocardiographic and hemodynamic variables between patient groups (mean ± sem).

	CRT	MM	*p* Value
Patient Number (N)	27	18	
All Patient Ages at Study (years)	23 ± 1.6	29 ± 3.0	<0.05
Number of Repaired CHD (N) (%)	16 (60%)	15 (100%)	<0.05
Patient Age at CHD Repair (mos)	27 ± 1.4	30 ± 3.3	NS
Existing V Arrhythmias (N) (%)	6 (22%)	6 (40%)	NS
Systemic RV (N) (%)	8 (30%)	5 (33%)	NS
Single V (N) (%)	2 (7%)	2 (13%)	NS
Patients pre-existing V pace (N) (%)	17 (63%)	12 (80%)	NS
Years V paced before HT listing (N)	12 ± 2	11 ± 3	NS
Baseline QRS duration (ms)	156 ± 5.7	149 ± 10.8	NS
Baseline EF (%)	31 ± 3.0	38 ± 3.7	NS
Baseline LVEDD (cm)	6.1 ± 0.2	5.4 ± 0.4	NS
Baseline V contractility (mmHg-s)	600 ± 32	666 ± 37	NS
Baseline V EDP (mmHg)	14.9 ± 1.0	14.2 ± 1.7	NS

Legend: Abbreviations as in text. As indicated, measured variables did not differ between groups. NS: non-significant.

## Data Availability

The original contributions presented in this study are included in the article. Further inquiries can be directed to the corresponding author.
